# Supplementary material for: Encapsulation Reduces the Deleterious Effects of Salicylic Acid Treatments on Root Growth and Gravitropic Response
Source: Int J Mol Sci. 2022 Nov 14;23(22):14019. doi: 10.3390/ijms232214019 (PMC9696185; doi:10.3390/ijms232214019)
Supplement: Supplementary file 1 [file ijms-23-14019-s001.zip › ijms-1971016-supplementary.pdf]

Supplementary Tables and Figures

Table S1. Root length of Col-0 Arabidopsis plants treated with free SA, Si:SA and Ch:SA. 5-day-old plants were transferred to media containing the different SA treatments and root length measured daily. Different letters indicate significant differences among treatment groups at $p \leq 0.05$.

	TREATMENTS					
		Doses				
**Days**	**Control**	**1 µM**	**10 µM**	**50 µM**	**100 µM**	**500 µM**
			**SA**			
**1**	0.70 ± 0.07^{a}	0.60 ± 0.10^{ab}	0.57 ± 0.15^{b}	0.59 ± 0.11^{b}	0.54 ± 0.11^{bc}	0.53 ± 0.09^{c}
**2**	1.16 ± 0.09^{a}	0.89 ± 0.11^{bc}	0.88 ± 0.09^{c}	0.77 ± 0.10^{d}	0.64 ± 0.20^{de}	0.57 ± 0.10^{e}
**3**	1.64 ± 0.14^{a}	1.19 ± 0.18^{bc}	1.08 ± 0.25^{c}	0.80 ± 0.16^{de}	0.69 ± 0.23^{e}	0.61 ± 0.13^{f}
**4**	2.06 ± 0.16^{a}	1.36 ± 0.19^{b}	1.16 ± 0.25^{c}	0.84 ± 0.15^{d}	0.70 ± 0.21^{de}	0.61 ± 0.12^{e}
**5**	2.39 ± 0.19^{a}	1.49 ± 0.22^{bc}	1.19 ± 0.27^{c}	0.87 ± 0.16^{d}	0.69 ± 0.22^{e}	0.58 ± 0.13^{f}
			**Si:SA**			
**1**	0.70 ± 0.07^{a}	0.60 ± 0.10^{ab}	0.64 ± 0.07^{ab}	0.56 ± 0.09^{ab}	0.61 ± 0.07^{ab}	0.53 ± 0.09^{bcd}
**2**	1.16 ± 0.09^{a}	1.06 ± 0.11^{ab}	1.03 ± 0.10^{ab}	0.95 ± 0.11^{b}	0.97 ± 0.07^{cd}	0.72 ± 0.14^{d}
**3**	1.64 ± 0.14^{a}	1.57 ± 0.18^{ab}	1.46 ± 0.19^{ab}	1.35 ± 0.11^{b}	1.28 ± 0.09^{c}	0.84 ± 0.20^{d}
**4**	2.06 ± 0.16^{a}	1.92 ± 0.20^{a}	1.79 ± 0.25^{ab}	1.54 ± 0.09^{c}	1.42 ± 0.08^{de}	0.92 ± 0.17^{e}
**5**	2.39 ± 0.19^{a}	2.13 ± 0.19^{a}	1.97 ± 0.21^{ab}	1.65 ± 0.08^{b}	1.48 ± 0.13^{c}	0.95 ± 0.18^{d}
			**Ch:SA**			
**1**	0.70 ± 0.07^{a}	0.60 ± 0.05^{ab}	0.55 ± 0.10^{ab}	0.59 ± 0.13^{ab}	0.60 ± 0.08^{ab}	0.57 ± 0.08^{ab}
**2**	1.16 ± 0.09^{a}	1.11 ± 0.09^{a}	0.97 ± 0.09^{ab}	1.03 ± 0.14^{ab}	0.95 ± 0.17^{b}	0.79 ± 0.11^{c}
**3**	1.64 ± 0.14^{a}	1.65 ± 0.13^{a}	1.56 ± 0.08^{ab}	1.45 ± 0.23^{b}	1.28 ± 0.25^{c}	0.93 ± 0.17^{d}
**4**	2.06 ± 0.16^{a}	2.16 ± 0.18^{a}	2.03 ± 0.17^{ab}	1.75 ± 0.32^{b}	1.50 ± 0.30^{c}	0.95 ± 0.19^{d}
**5**	2.39 ± 0.19^{a}	2.52 ± 0.26^{a}	2.29 ± 0.20^{a}	1.94 ± 0.40^{ab}	1.56 ± 0.27^{b}	1.04 ± 0.18^{c}

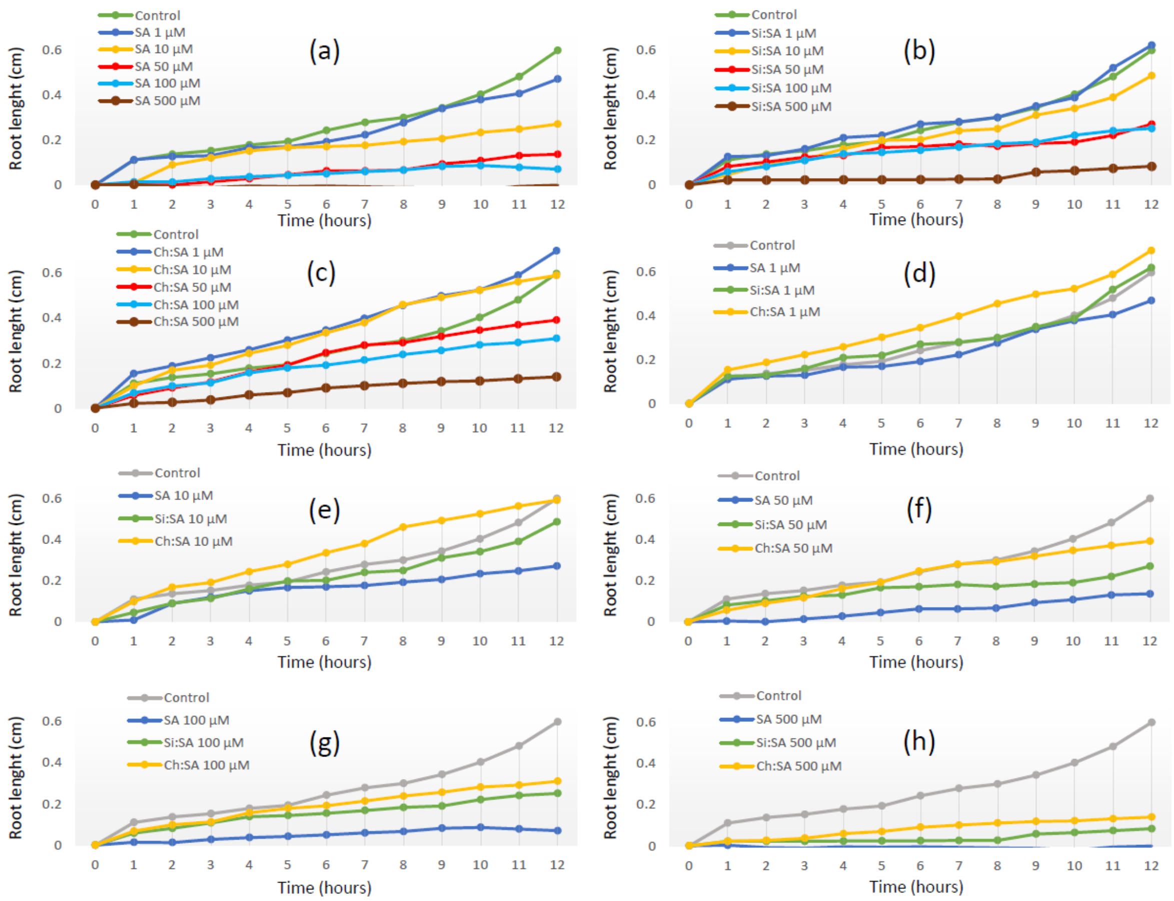

Figure S1. Effect of free SA, Si:SA and Ch:SA on root growth in Col-0 Arabidopsis plants. 5-day-old plants were transferred to media containing the different SA treatments and root length measured each hour (0 to 12 hours). Graphs (a), (b) and (c) compare root length among the doses at each treatment, and graphs (d), (e), (f), (g) and (h) compare root length among the treatments at each dose.

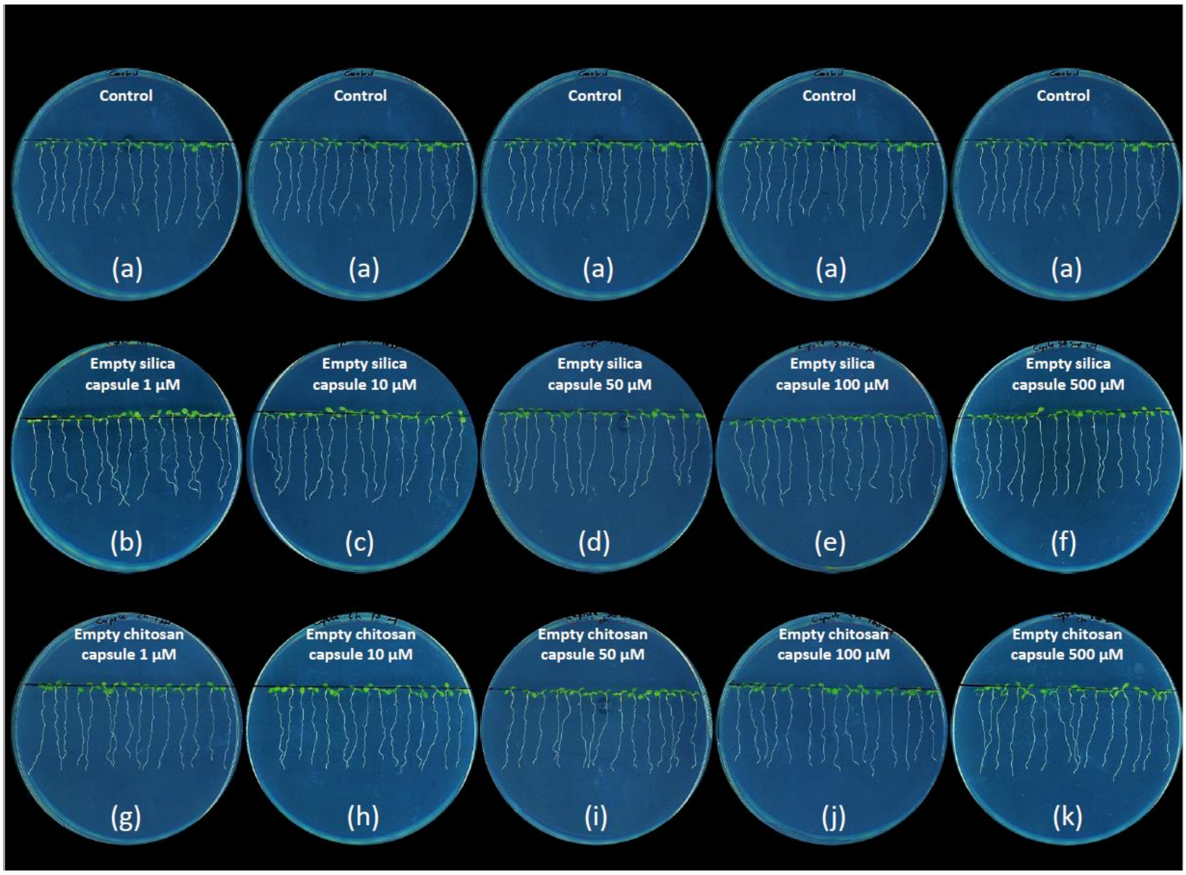

Figure S2. Effect of empty silica and chitosan capsules on root growth in Col-0 Arabidopsis plants. 5-day-old plants were transferred to media containing the empty capsules and pictures were taken 5 days later.

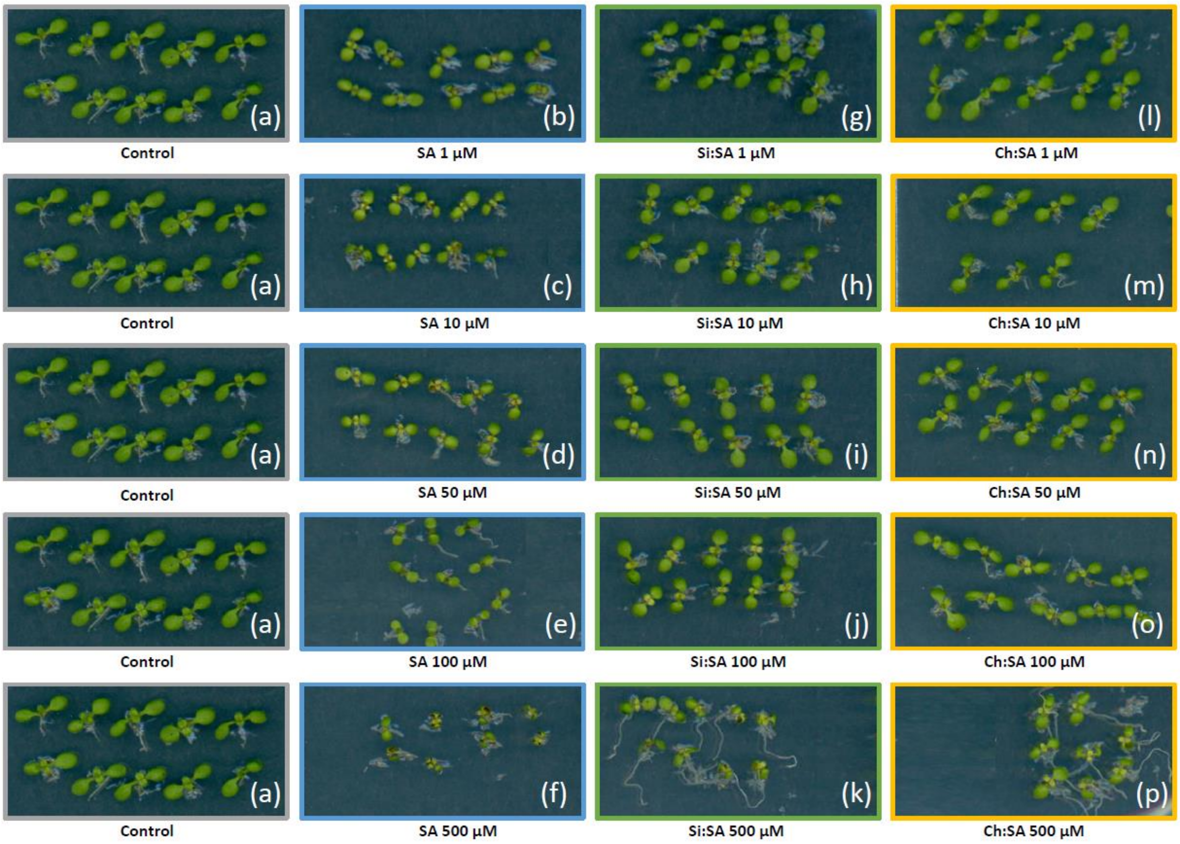

Figure. S3. Effect of free SA, Si:SA and Ch:SA on rosette size in Col-0 Arabidopsis plants. 5-day-old plants were transferred to media containing the different SA treatments and pictures were taken 5 days later.

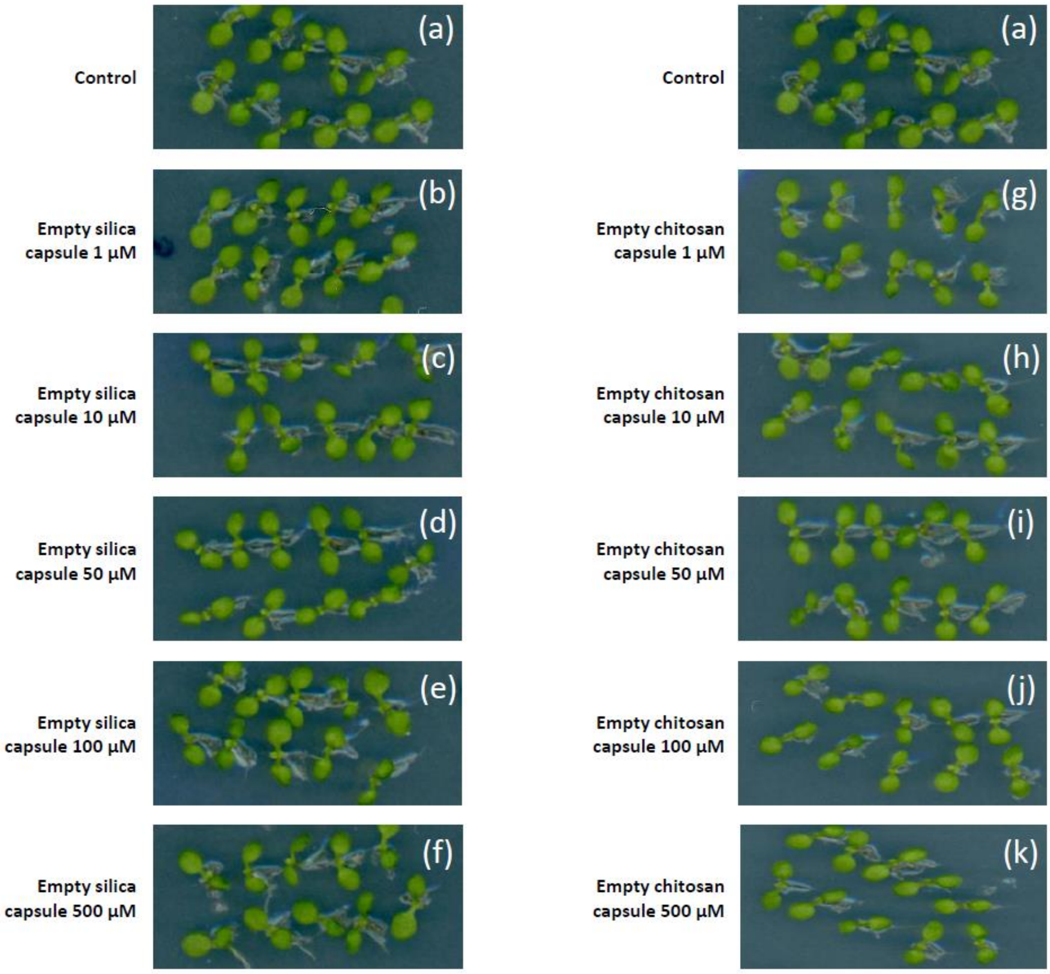

Figure. S4. Effect of empty silica and chitosan capsules on rosette size in Col-0 Arabidopsis plants. 5-day-old plants were transferred to media containing the empty capsules and pictures were taken 5 days later.

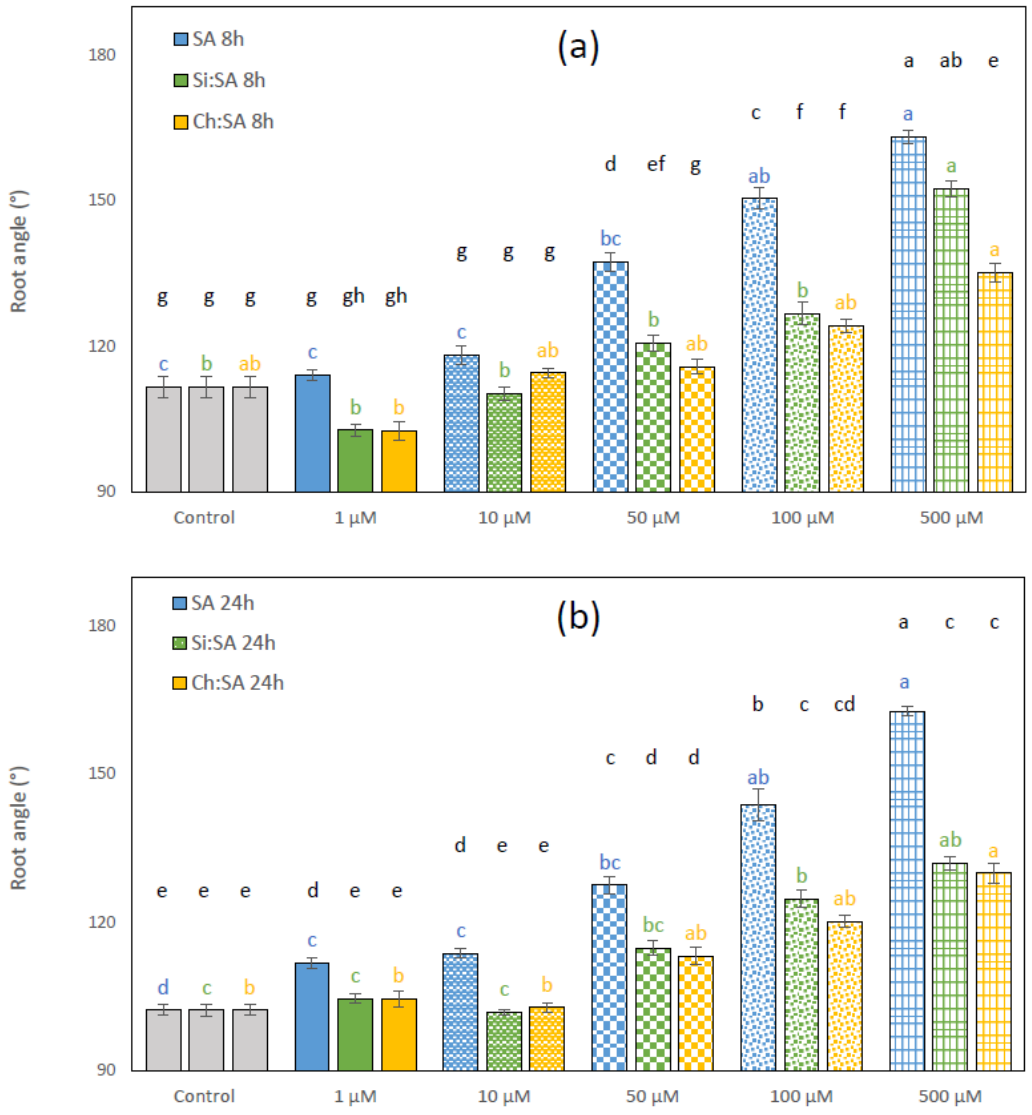

Figure.S5. Effect of free SA, Si:SA and Ch:SA on root reorientation in Col-0 Arabidopsis plants. 5-day-old plants were transferred to media containing the different SA treatments and root angle measured 8 and 24 hours later. Different letters indicate significant differences among treatment groups at $p \leq 0.05$. The colored letters show the differences between dose and Control in that particular treatment, and the black letters show the differences between dose and Control among all treatments.

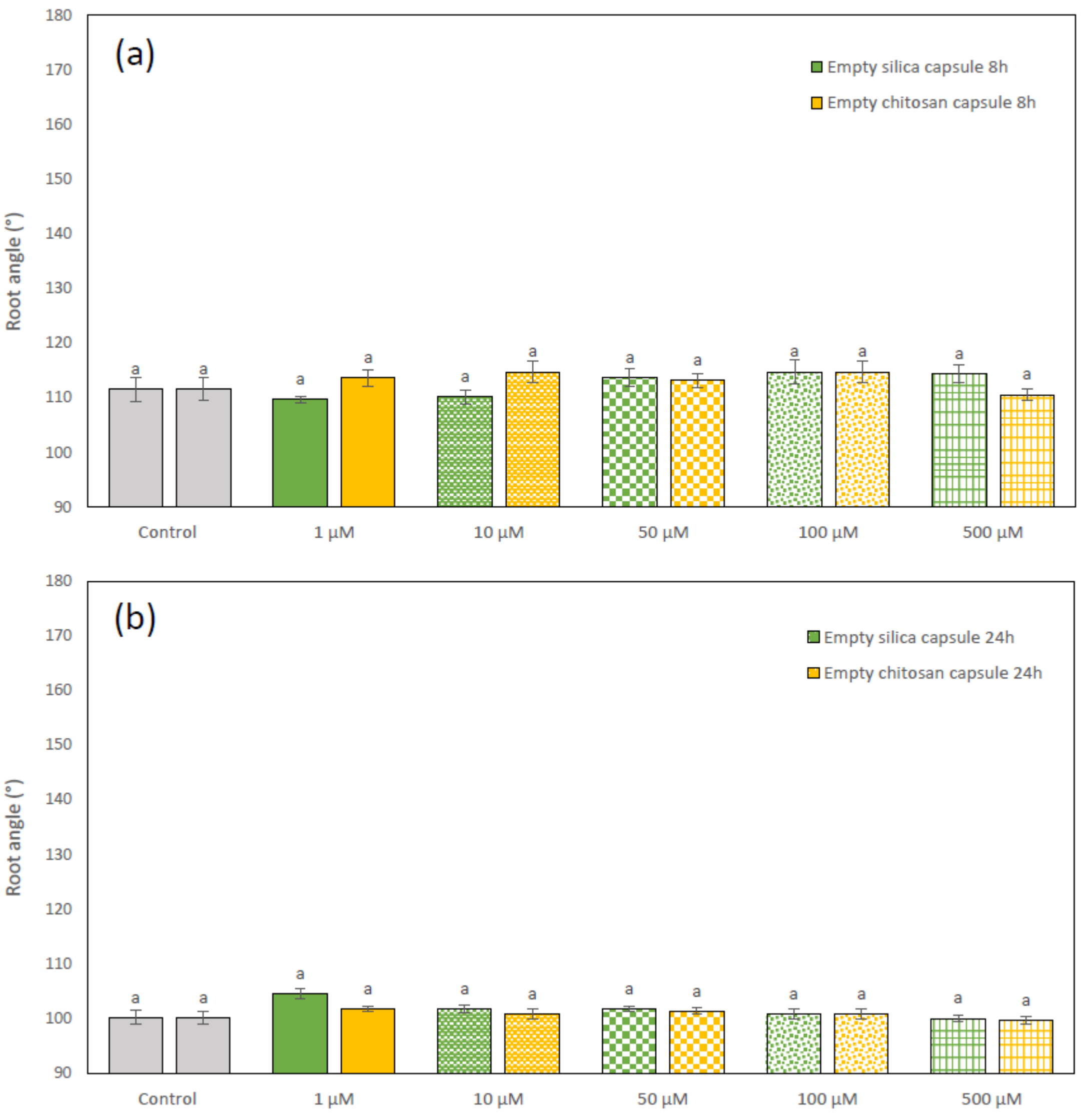

Figure.S6. Effect of empty silica and chitosan capsules on root reorientation in Col-0 Arabidopsis plants. 5-day-old plants were transferred to media containing the empty capsules and root angle measured 8 and 24 hours later. Different letters indicate significant differences among treatment groups at $p \leq 0.05$.

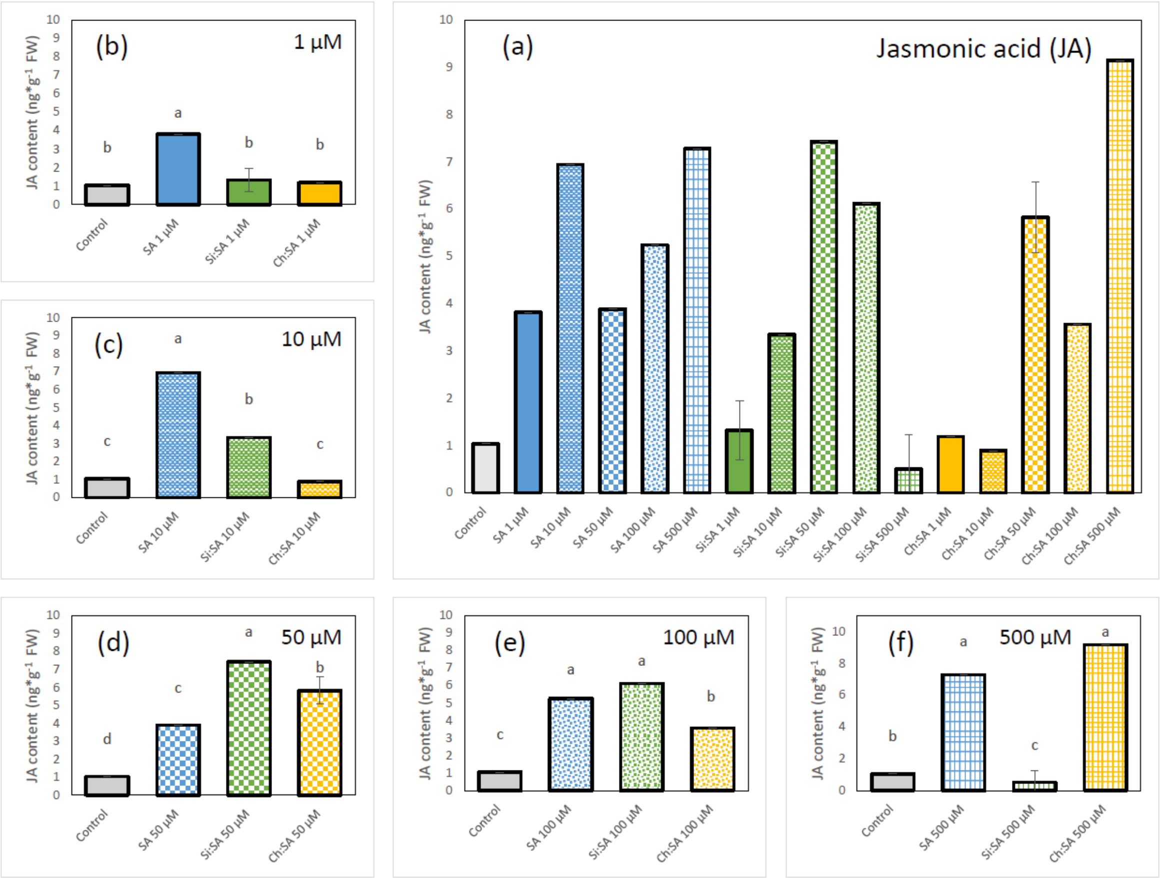

Figure. S7. Effect of free SA, Si:SA and Ch:SA on endogenous JA levels in roots of Col-0 Arabidopsis plants. 5-day-old plants were transferred to media containing the different SA treatments and plant hormones measured 28 days later. Graph (a) depicts JA levels in the three treatments at all doses, and graphs (b), (c), (d), (e) and (f) compare JA levels among the treatments at each dose. Different letters indicate significant differences among treatment groups at $p \leq 0.05$.

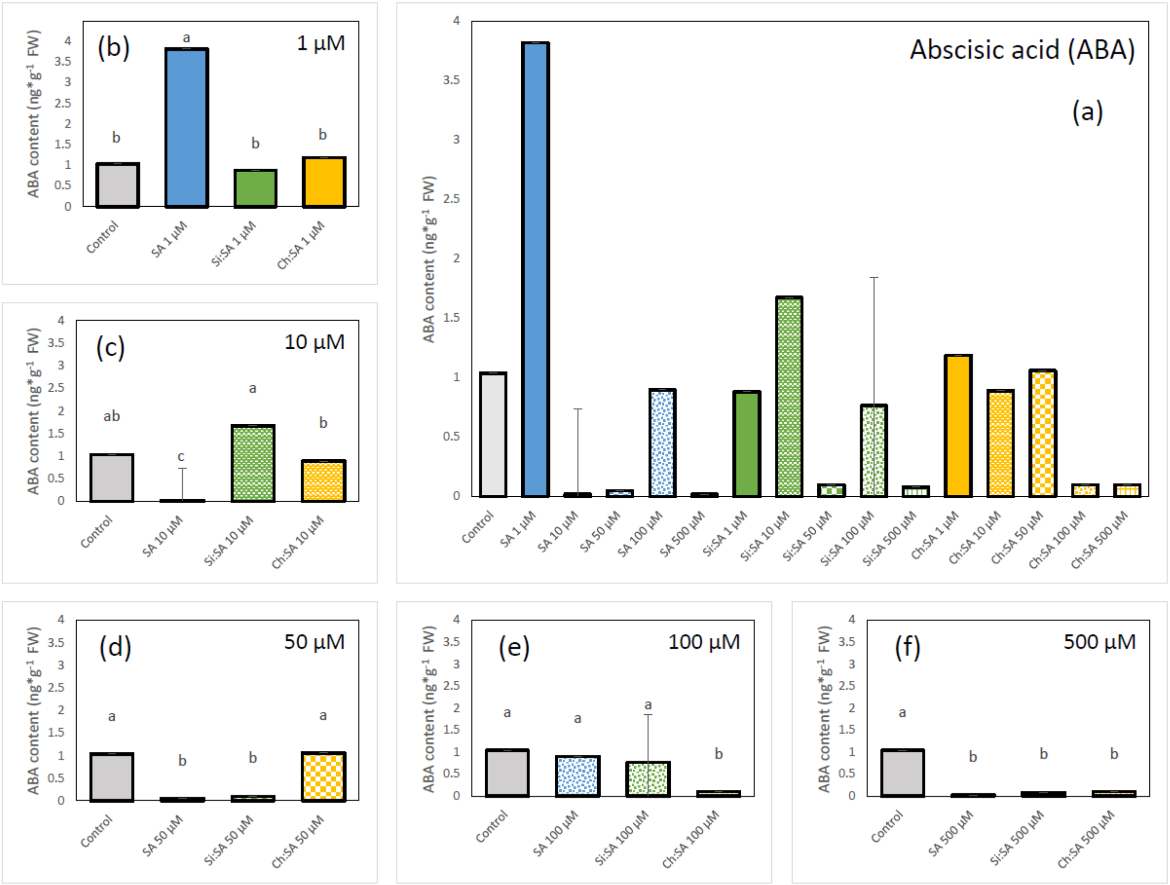

Figure. S8. Effect of free SA, Si:SA and Ch:SA on endogenous ABA levels in roots of Col-0 Arabidopsis plants. 5-day-old plants were transferred to media containing the different SA treatments and plant hormones measured 28 days later. Graph (a) depicts ABA levels in the three treatments at all doses, and graphs (b), (c), (d), (e) and (f) compare ABA levels among the treatments at each dose. Different letters indicate significant differences among treatment groups at $p \leq 0.05$.

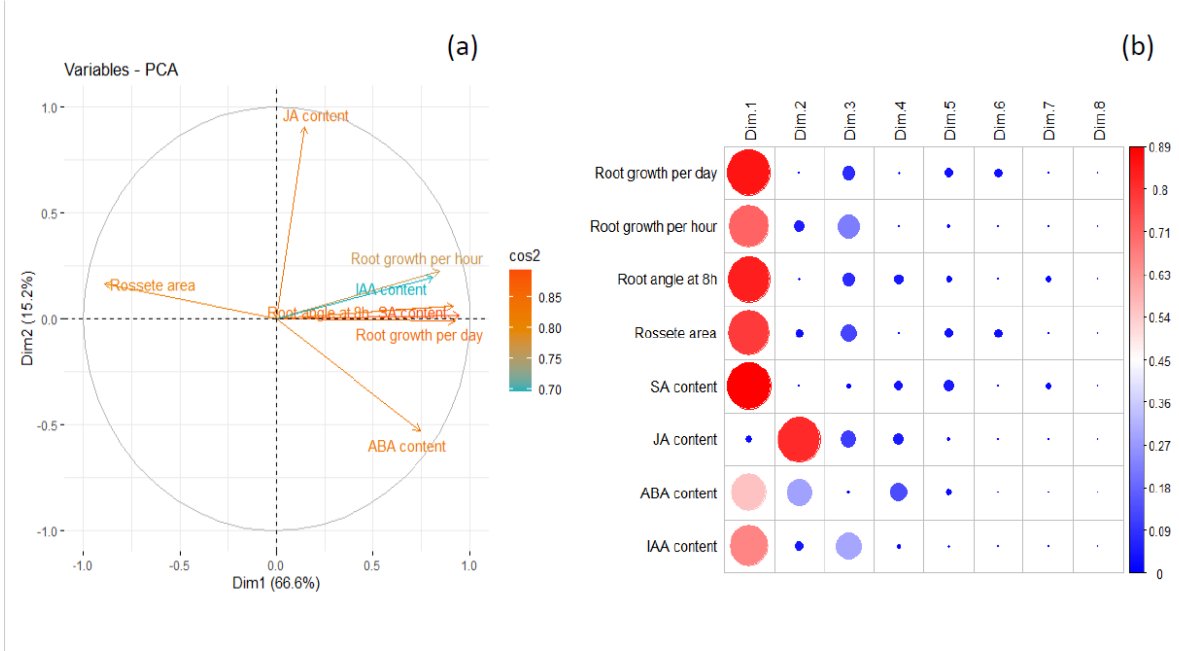

Figure. S9. Correlation graphics of variables measured in Col-0 Arabidopsis plants treated with free SA, Si:SA and Ch:SA at 1, 10, 50, 100 and 500 µM doses. Graph (a) depicts the Variables-PCA plot where, in the cos2 color scale, red denotes a good representation of the variable in the principal component and blue that the variable is not perfectly represented, and graph (b) depicts correlation variables matrix.
